# Potential diagnostic biomarkers: 6 cuproptosis- and ferroptosis-related genes linking immune infiltration in acute myocardial infarction

**DOI:** 10.1038/s41435-023-00209-8

**Published:** 2023-07-08

**Authors:** Mengdan Miao, Shanhu Cao, Yifei Tian, Da Liu, Lixia Chen, Qiaoying Chai, Mei Wei, Shaoguang Sun, Le Wang, Shuanli Xin, Gang Liu, Mingqi Zheng

**Affiliations:** 1 Department of Cardiology, The First Hospital of Hebei Medical University, Shijiazhuang, 050031 Hebei China; 2 Hebei Key Laboratory of Heart and Metabolism, Shijiazhuang, 050000 Hebei China; 3 Department of Cardiology, Handan First Hospital, Handan, 056000 Hebei China; 4 Department of Biochemistry and Molecular Biology, Hebei Medical University, 050017 Shijiazhuang, China

**Keywords:** Gene expression, Epigenomics

## Abstract

The current diagnostic biomarkers of acute myocardial infarction (AMI), troponins, lack specificity and exist as false positives in other non-cardiac diseases. Previous studies revealed that cuproptosis, ferroptosis, and immune infiltration are all involved in the development of AMI. We hypothesize that combining the analysis of cuproptosis, ferroptosis, and immune infiltration in AMI will help identify more precise diagnostic biomarkers. The results showed that a total of 19 cuproptosis- and ferroptosis-related genes (CFRGs) were differentially expressed between the healthy and AMI groups. Functional enrichment analysis showed that the differential CFRGs were mostly enriched in biological processes related to oxidative stress and the inflammatory response. The immune infiltration status analyzed by ssGSEA found elevated levels of macrophages, neutrophils, and CCR in AMI. Then, we screened 6 immune-related CFRGs (CXCL2, DDIT3, DUSP1, CDKN1A, TLR4, STAT3) to construct a nomogram for predicting AMI and validated it in the GSE109048 dataset. Moreover, we also identified 5 pivotal miRNAs and 10 candidate drugs that target the 6 feature genes. Finally, RT-qPCR analysis verified that all 6 feature genes were upregulated in both animals and patients. In conclusion, our study reveals the significance of immune-related CFRGs in AMI and provides new insights for AMI diagnosis and treatment.

## Introduction

Acute myocardial infarction (AMI), a myocardial injury event induced by the rupture of atherosclerotic plaques and thrombosis [1], is a primary cause of mortality and disability in cardiovascular disease across the world [2]. Rapid and accurate identification of AMI is essential for early interventional treatment and improvement of prognosis. Elevated troponin is one of the diagnostic gold standards for AMI. However, the specificity of troponin is often limited, as it can also be elevated in myocarditis, cardiomyopathy, and other non-cardiac diseases such as renal failure, severe sepsis, and so on [3, 4]. Therefore, it needs to identify novel and more precise biomarkers for diagnosing AMI.

Previous studies have shown that immune cells play crucial roles in plaque progression [5], including foam cell formation, vascular smooth cell migration [6], and fibrous cap degradation, leading to plaque instability and increased risk of rupture and thrombosis, resulting in acute myocardial infarction (AMI) [7]. Immune cells also play vital roles in post-AMI injury repair, such as clearing debris, forming infarct area, and promoting vascular reconstruction [8].

Ferroptosis is an iron-dependent form of programmed cell death with a distinct mechanism from autophagy, necrosis, apoptosis, and pyroptosis [9]. Mitochondrial atrophy, increased membrane density, iron accumulation, and lipid peroxidation are characteristics of ferroptosis [10]. There is considerable evidence that ferroptosis mediates the development of several cardiovascular diseases, including atherosclerosis, myocardial infarction, heart failure, and cardiomyopathy [11, 12]. During AMI, the downregulation of GPX4 (a key regulator of ferroptosis) contributes to cardiomyocyte ferroptosis and myocardial injury [13]. While the exosomes of MSCs derived from human umbilical cord blood exhibit cardioprotective advantages by preventing ferroptosis in AMI mouse models [14]. Moreover, ferroptosis could promote inflammation and aggravate the cardiac dysfunction and poor ventricular remodeling after AMI [15]. Meanwhile, several studies have revealed that ferroptosis-related genes expressed specifically in the peripheral blood of AMI patients may be potential biomarkers for predicting AMI [16, 17]. In addition, another novel kind of programmed cell death, cuproptosis, has also been demonstrated to contribute to the development of AMI.

Excessive copper accumulation in mitochondria leads to cuproptosis, a form of cell death characterized by lipoylated protein accumulation and reduced Fe-S cluster proteins [18]. Recent studies have linked cuproptosis to AMI, shedding new light on diagnosis and therapy [19]. Intracellular copper overload has been identified as a primary contributor to cuproptosis, and its connection with ferroptosis has been observed in other studies [20]. Diagnostic models for AMI based on cuproptosis- or ferroptosis-related genes have been proposed separately [17–19], but AMI pathophysiology involves complex molecular mechanisms. Combining cuproptosis, ferroptosis, and immune infiltration analysis may enable more precise identification of diagnostic biomarkers. In this study, we systematically analyzed the expression of cuproptosis- and ferroptosis-related genes (CFRGs) and their relationship with immune infiltration in AMI patients, identified 6 feature genes, and constructed a diagnostic model. RT-qPCR validation was performed in AMI mouse models and patients, providing novel diagnostic ideas for AMI.

## Materials and methods

### Datasets collection

Four gene expression profiles (GSE48060, GSE66360, GSE97320, GSE62646) were downloaded from Gene Expression Omnibus (GEO). The training datasets (GSE48060, GSE66360, and GSE97320) were generated using GPL570-Affymetrix Human Genome U133 plus 2.0 Array [HG-U133_Plus_2]. The GSE62646 dataset was used as the validation dataset and generated using GPL6244-Affymetrix Human Gene 1.0 ST Array [HuGene-1_0-st]. GSE48060 contained peripheral blood samples from patients (*n* = 31) with AMI and controls (*n* = 21) with normal cardiac function. The GSE66360 dataset included peripheral blood samples from 49 AMI patients and 50 healthy individuals. The GSE97320 dataset consisted of peripheral blood samples from three healthy controls and three AMI patients. In addition, peripheral blood samples from 14 healthy volunteers and 28 patients following the first day of AMI were screened from the GSE62646 dataset.

### Data preparation and the **CFRGs** identification

The three raw datasets were transformed into an expression value matrix using the “limma” package in the R software (Version 4.2.0) [21], The batch effects were removed using the “sva” package after merging the three datasets (GSE48060, GSE66360, GSE97320) [22]. The ferroptosis-related genes (FRGs) were obtained from the FerrDb website (http://www.zhounan.org/ferrdb/) [23]. The cuproptosis-related genes (CRGs) were collected from a previous study [18]. The Pearson correlation coefficients (PCCs) of the CRGs and FRGs expression levels were calculated using the “correlation test function (cor.test())” in R [24], the threshold was set as “|cor | >0.3 and *p* < 0.05”, and a total of 175 CFRGs were identified.

### Functional enrichment analysis

The differential CFRGs were identified by the “limma” R package with the threshold “|log2FC | >0.2 and FDR < 0.05” [25]. The results were visualized as volcano plot and heatmap using the “ggplot2” and “pheatmap” R packages. Subsequently, functional enrichment analysis of differential CFRGs, including Gene Ontology (GO) and Kyoto Encyclopedia of Genes and Genomes (KEGG), were performed using the “clusterProfiler” R package [26]. The pathways with *P* < 0.05 were identified as significant.

### PPI network construction and hub CFRGs identification

The PPI network of the differential CFRGs was constructed via the STRING database (https://www.string-db.org/) with the cutoff interaction score set at 0.4. Then the top 10 hub CFRGs with the highest maximal clique centrality (MCC) values were selected via the "Cytohubba" plugin of Cytoscape and visualized using Cytoscape software (version 3.9.1).

### Infiltration analysis of immune cells and functions

The infiltrating score of 16 immune cells and the activity of 13 immune functions in healthy and AMI groups were calculated with single-sample gene set enrichment analysis (ssGSEA) via the “gsva” R package [13] and visualized by heatmap using the “pheatmap” package [27]. The violin plots were used to compare and visualize the ssGSEA scores of infiltrated immune cells and functions between the healthy and AMI samples by “ggpubr” R package [28]. The correlation heatmap, which revealed the correlation of 29 types of immune cells and related functions, was performed using the “corrplot” R package.

### Construction and verification of the diagnostic model

To screen the CFRGs with diagnostic potential, we analyzed the relationship among 10 hub CFRGs with immune cells and functions using Spearman’s correlation analysis via the “ggcorrplot” R package, and identified 6 feature genes (CXCL2, DDIT3, DUSP1, CDKN1A, TLR4, STAT3) with significant correlations with immune infiltration for predicting AMI. Then the 6 feature genes were subjected to Logistic regression analysis to construct a nomogram model via the “rms” R package. To evaluate the diagnostic performance of feature genes, the ROC was plotted using the “pROC” R package. Subsequently, the 6 feature genes were verified by the GSE62646 validation dataset.

### Identification of pivotal miRNAs and candidate drugs

The pivotal miRNAs targeting the 6 feature genes were predicted with the Targetscan database [29], and the candidate drugs were determined using the DSigDB database. The access of the Targetscan database and DSigDB database are acquired through Enrichr (http://amp.pharm.mssm.edu/Enrichr/) platform.

### Acute myocardial infarction model created

Male C57BL/6 mice (8 weeks old) were obtained from Shandong Hengrong Bio-Technology Co., Ltd (Jinan, China) and randomly assigned to either the AMI group or the sham group. The randomization process was conducted using a random number table. Mice were housed in a temperature-controlled environment with 12 h light/12 h dark cycles and allowed to acclimate for 1 week before surgery. Under isoflurane anesthesia, left pectoralis major and pectoralis minor muscles were separated to expose the heart, and the left anterior descending (LAD) coronary artery was ligated to induce regional ischemia, confirmed by ECG alterations. Sham surgery involved the same procedure but without LAD ligation. Echocardiography was performed 24 h post-myocardial infarction to measure changes in left ventricular ejection fraction (LVEF) and fractional shortening (LVFS). After euthanasia, cardiac tissue and peripheral blood were harvested. The animal study protocol was approved by the Animal Care and Use Committee of the First Hospital of Hebei Medical University and complied with NIH regulations (Guide for the Care and Use of Laboratory Animals).

### Collecting peripheral blood from AMI patients and healthy controls

This is a prospective study that included 20 AMI patients and 20 healthy controls, with no age or gender restrictions. All AMI patients were recruited from the Department of Cardiology at the First Hospital of Hebei Medical University, and they all met the 2017 European Society of Cardiology (ESC) ST-segment elevation myocardial infarction (STEMI) criteria [30], including typical symptoms of myocardial ischemia; the ECG must show ST-segment elevation of at least 1 mm in two or more contiguous leads or new-onset left bundle branch block; the biomarker of myocardial necrosis must be elevated above the 99th percentile of the upper reference limit; coronary angiography shows stenosis or occlusion. Patients with Non-ST-segment elevation myocardial infarction (NSTEMI) or Unstable angina (UA) were excluded. Peripheral blood (5 mL) was collected from patients within 24 h of the onset of myocardial infarction. The healthy individuals were selected from the Health Examination Center as controls, which were matched in gender and similar age with the AMI patients. Peripheral blood samples (5 ml) were collected in the morning after an overnight fast. This research was conducted in compliance with the principles of the Helsinki Declaration and with the approval of the First Hospital of Hebei Medical University’s Ethics Committee.

### RNA isolation and quantitative real-time PCR

Total RNA of peripheral blood and heart tissue were extracted using chloroform and TRIpure Reagent LS (Aidlab, RN0202). Hifair® III 1st Strand cDNA Synthesis SuperMix (Yeasen,11141ES10) was used to reverse transcribe cDNA. Gene expression was examined with a Roche480 LightCycler® 96 real-time PCR System by a qPCR SYBR Green Master Mix (Yeasen, 11184ES03). Samples were run on triplicate plates and their Cq values averaged. Relative quantitation was performed using 2^−△△^Cq method. Data were performed using GAPDH as a reference gene. All qPCR primers used are listed in Supplementary Table 1.

### Statistical analysis

The statistical analysis was performed using R software(version4.2.0), GraphPad Prism (Version 9.0), and SPSS (Version 23.0). Continuous variables were expressed as mean ± SD or median (quartile). The Student’s *t* test and Mann–Whitney test were used to test continuous variables with or without normal distribution, respectively. Categorical variables were presented as numbers (percentages) by using the chi-square test. The statistical significance was set at *P* < 0.05 (two-sided).

## Results

### Differential CFRGs between AMI patients and healthy controls

**Fig. 1:**
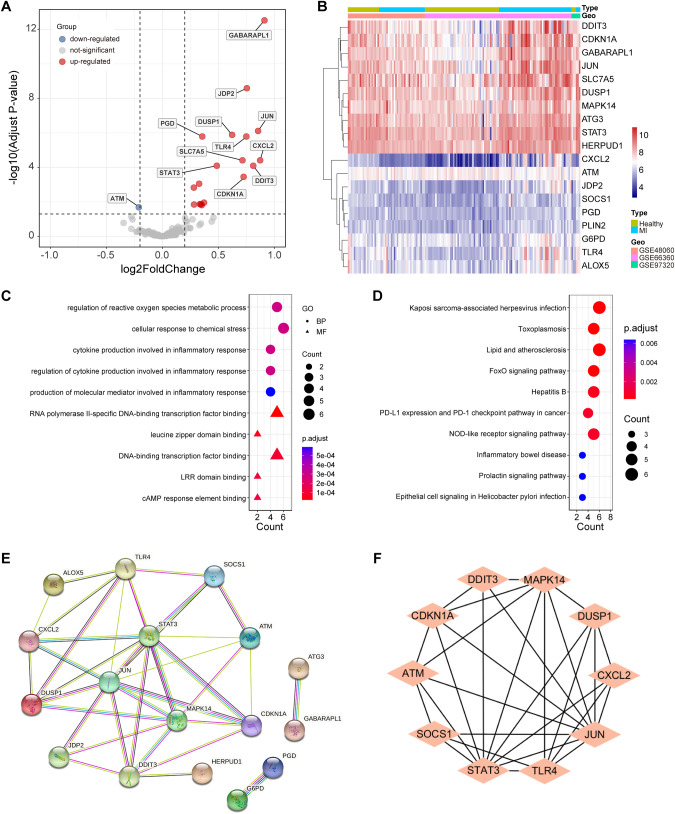
GO/KEGG analysis and the PPI network of differential CFRGs. **A** Volcano plot of the differential CFRGs from the combined datasets. **B** Heatmap of the differential CFRGs from the combined datasets in AMI patients and healthy controls. **C** GO analysis of differential CFRGs. **D** KEGG analysis of differential CFRGs. **E** PPI network of the differential CFRGs. **F** Identification of 10 hub CFRGs.

We obtained 74 healthy controls and 83 AMI samples data from three datasets (GSE48060, GSE66360, and GSE97320) in GEO. The expression data were normalized and visualized by box plots in Supplementary Fig. 1A, B. The batch effect was corrected through the PCA algorithm (Supplementary Fig. 2A, B). A total of 13 CRGs (FDX1, LIPT1, LIAS, DLD, DBT, GCSH, DLST, DLAT, PDHA1, PDHB, SLC31 A1, ATP7A, ATP7B) was obtained from the previous study, and 259 FRGs were identified from the FerrDb website after removing the duplicated genes from the three subgroups (drivers, suppressors, and features). The detailed information and classification of FRGs were attached in Supplementary Table 3. Then, through the Pearson correlation analysis, 175 CFRGs were obtained using the “cor.test()” function of R. The detailed information of their PCCs is attached in Supplementary Table 4. Finally, based on the criterion |log2FC | > 0.2 and FDR < 0.05, 19 differential CFRGs were identified, 18 of which were upregulated and 1 downregulated, and visualized by the volcano plot and heatmap (Fig. 1A, B).

### Functional analysis of the differential CFRGs

Functional enrichment analysis indicated that the differential CFRGs were mainly involved in oxidative stress and inflammatory response biological process (BP) terms, including “regulation of reactive oxygen species metabolic process”, “cellular response to chemical stress”, “cytokine production involved in inflammatory response”, “regulation of cytokine production involved in inflammatory response” and “production of molecular mediator involved in inflammatory response” (Fig. 1C). The molecular function (MF) analysis enriched in “RNA polymerase II-specific DNA-binding transcription factor binding”, “leucine zipper domain binding”, “DNA-binding transcription factor binding”, “LRR domain binding” and “cAMP response element binding” (Fig. 1C). In addition, the KEGG pathway of differential CFRGs was found to be enriched in “Kaposi sarcoma-associated herpesvirus infection”, “Toxoplasmosis”, “Lipid and atherosclerosis”, “FoxO signaling pathway”, “Hepatitis B”, “PD-L1 expression and PD-1 checkpoint pathway in cancer”, “NOD-like receptor signaling pathway”, “Inflammatory bowel disease”, “Prolactin signaling pathway” and “Epithelial cell signaling in Helicobacter pylori infection” (Fig. 1D).

### Identification of hub CFRGs

The protein-protein interaction was obtained from the STRING database. After removing the isolated nodes, the PPI network was constructed with 17 nodes and 37 edges, including 16 upregulated and 1 downregulated differential CFRGs, respectively (Fig. 1E). Finally, the top 10 hub CFRGs, including JUN, STAT3, MAPK14, TLR4, CDKN1A, DUSP1, DDIT3, ATM, CXCL2, and SOCS1, were identified by "Cytohubba" via the Cytoscape software (Fig. 1F).

### Infiltration analysis of immune cells and functions

**Fig. 2:**
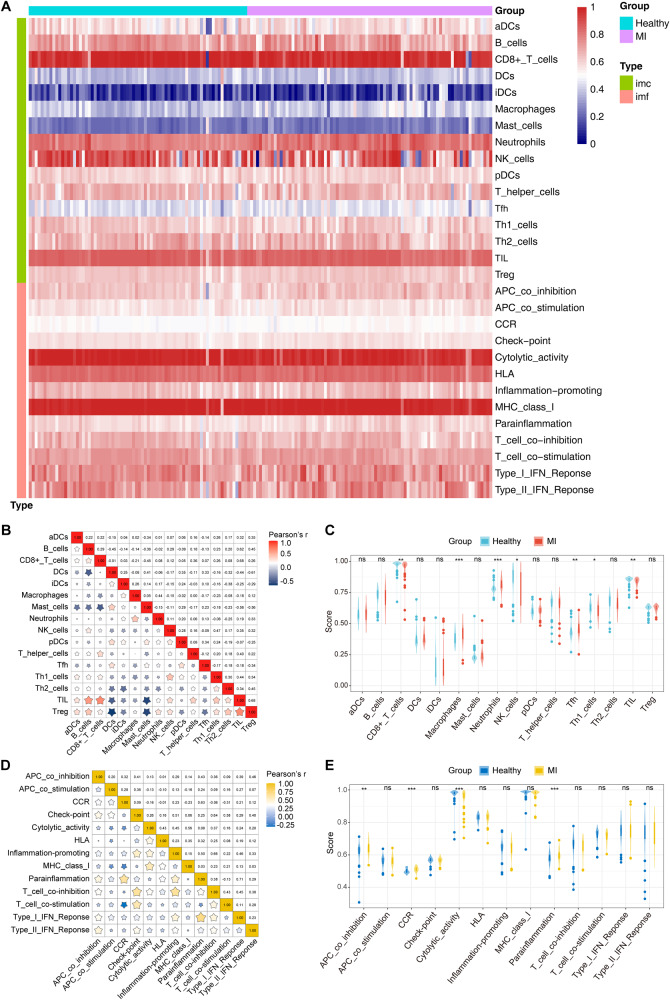
Differentially infiltrated immune cells and functions in AMI patients and healthy controls. **A** Heatmap of differential immune cells and functions. **B** Correlation matrix of 16 immune cells. **C** The ssGSEA scores of 16 immune cells. **D** Correlation matrix of 13 immune functions. **E** The ssGSEA scores of 13 immune functions. CCR, cytokine-cytokine receptor. * *P* < 0.05, ** *P* < 0.01, *** *P* < 0.001.

To further investigate the infiltration of immune cells and functions between AMI patients and healthy controls, we assessed the enrichment scores of distinct immune cell subpopulations and functions using ssGSEA. The results were visualized via the heatmap (Fig. 2A) and violin plots (Fig. 2C, E). AMI patients showed elevated levels of macrophages, neutrophils, Tfh cells, APC co inhibition, CCR, and parainflammation, but decreased levels of cytolytic activity and CD8 + T cells, NK cells, Th1 cells, and TIL cells. The correlation of 16 immune cells indicated that activated TIL cells were positively connected with B cells (*r* = 0.62), CD8 + T cells (*r* = 0.68), and Treg cells (*r* = 0.68), while activated Treg cells were negatively correlated with DCs (*r* = −0.61). In addition, activated mast cells were negatively correlated with TIL cells and Treg cells (*r* = −0.56) (Fig. 2B). The correlation of 13 types of immune functions revealed that activated parainflammation was positively correlated with Type I IFN-Response (r = 0.71) and CCR (*r* = 0.63), and that T cell co inhibition was positively correlated with checkpoint (*r* = 0.64) and inflammation promoting (*r* = 0.66) (Fig. 2D).

### Identification of 6 feature genes for diagnosing AMI

**Fig. 3:**
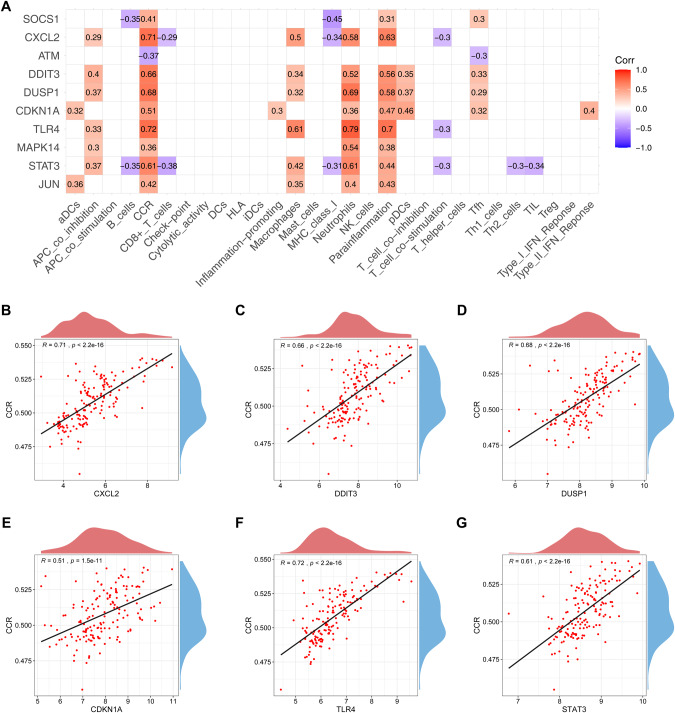
Correlation among hub CFRGs with immune cells and functions. **A** Heatmap of correlation among 10 hub CFRGs with immune cells and functions. Scatter diagram of the correlation between CCR and CXCL2 (**B**), DDIT3 (**C**), DUSP1 (**D**), CDKN1A (**E**), TLR4 (**F**), STAT3 (**G**).

To determine the diagnostic biomarkers for AMI, we analyzed the association of 10 hub CFRGs with immune cells and functions (Fig. 3A), and found a strong positive association between CCR and multiple genes, including CXCL2, DDIT3, DUSP1, CDKN1A, TLR4, STAT3. The correlation spot plots were plotted separately (Fig. 3B–G). In addition, DUSP1, TLR4, and STAT3 have also been observed to have a significant positive correlation with neutrophils. This indicates that the CCR and neutrophils play an important role in CFRGs pathways in AMI.

**Fig. 4:**
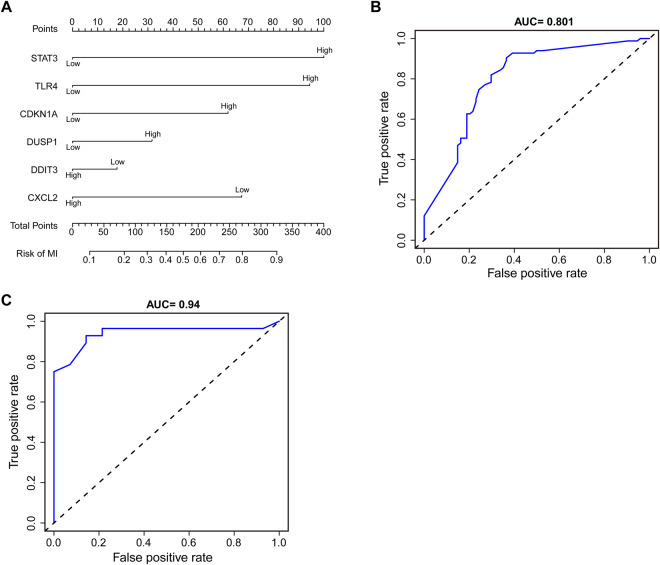
Diagnostic model construction. **A** Construction of a nomogram model with 6 feature genes. **B**, **C** ROC curve for evaluating and validating the diagnostic model’s performance.

Then, we used the 6 hub CFRGs (CXCL2, DDIT3, DUSP1, CDKN1A, TLR4, STAT3) most associated with immune infiltration as diagnostic feature genes for predicting AMI and constructing a nomogram (Fig. 4A). The ROC curve was used to evaluate the diagnostic performance of the 6-gene signature model, and the AUC value of the training dataset was 0.805 (Fig. 4B). The efficiency of the prediction model was validated by the GSE62646 dataset (AUC = 0.94) (Fig. 4C), indicating that the signature exhibited a good predictive value for AMI. However, the accuracy and reliability of this diagnostic model will need to be further investigated in future clinical trials.

### Identification of pivotal miRNAs and candidate drugs

**Fig. 5:**
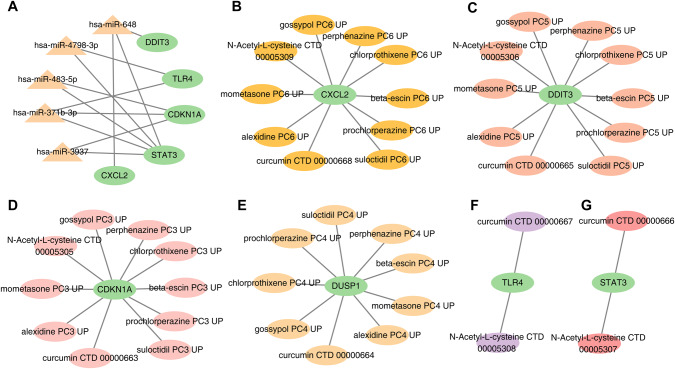
MiRNA-mRNA and drug-gene network construction. **A** 5 Pivotal miRNAs targeting 6 feature genes. **B**–**G** 10 candidate drug molecules targeting 6 feature genes.

To identify the pivotal miRNAs and candidate drugs targeting the 6 feature genes, the data was collected from the Targetscan database and DSigDB database. Ultimately, a total of 5 miRNAs were screened with a set *P* value < 0.05 (Fig. 5A). It was shown that CDKN1A interacts with three miRNAs: hsa-miR-371b-3p, hsa-miR-483-5p, and hsa-miR-3937. And the TLR4 interacted with the miRNAs hsa-miR-371b-3p and hsa-miR-4798-3p. However, STAT3 could be regulated by all 5 miRNAs (hsa-miR-371b-3p, hsa-miR-648, hsa-miR-4798-3p, hsa-miR-483-5p, hsa-miR-3937). But DDIT3 and CXCL2 have only been demonstrated to interact with hsa-miR-648.

In addition, we screened the top 10 drug molecules according to the set adjusted *p* value < 0.05 by the DSigDB database (Fig. 5B–G). Curcumin CTD 00000663, the drug molecule containing all 6 feature genes interactions, with a combined score of 2550948.8. N-Acetyl-L-cysteine CTD 00005305 was associated with 5 feature genes (CDKN1A; DDIT3; STAT3; TLR4; CXCL2). The remaining drug molecules have been indicated that interacted with CDKN1A, DUSP1, DDIT3 and CXCL2. These drug candidates also provide inspiration for the research and development of AMI treatment.

### AMI mouse model validation

**Fig. 6:**
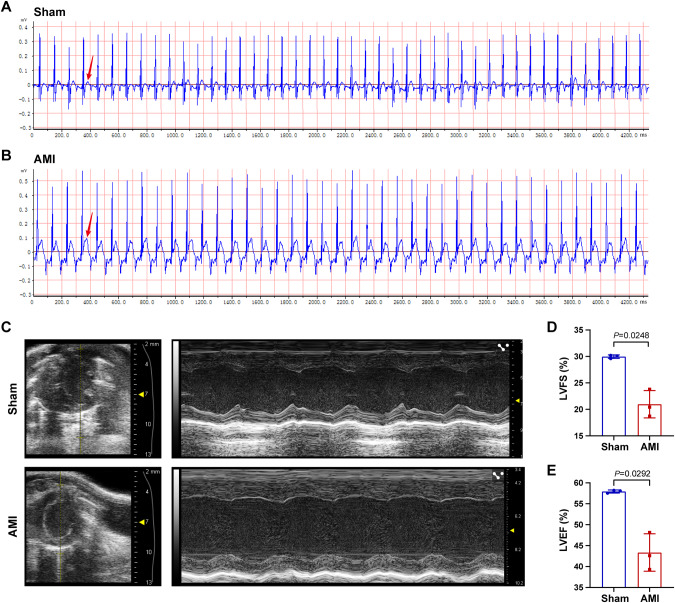
AMI mouse model validation. **A**, **B** Representative ECG images of AMI (*n* = 3) and sham animals (*n* = 3) were obtained after the cardiac surgery. The arrow noted that ST segment was elevated in AMI animals. **C** Representative echocardiography images of two groups at 24 h after the cardiac surgery. **D**, **E** Comparison of LVFS and LVEF in the two groups. ECG: electrocardiogram, LVFS: Left ventricular fractional shortening, LVEF: Left ventricular ejection fraction.

Following surgery, the ECGs of mice in the AMI and sham groups were measured (Fig. 6A, B), and ST-segment elevation was observed on the ECGs of AMI mice. After 24 h post-surgery, the LVEF and LVFS were evaluated by Doppler M-mode ultrasonography, as shown in Fig. 6C. The results have shown that LVFS and LVEF values were significantly lower in the AMI group than the sham (Fig. 6D, E).

### Validation of 6 feature genes using RT-qPCR analysis

**Fig. 7:**
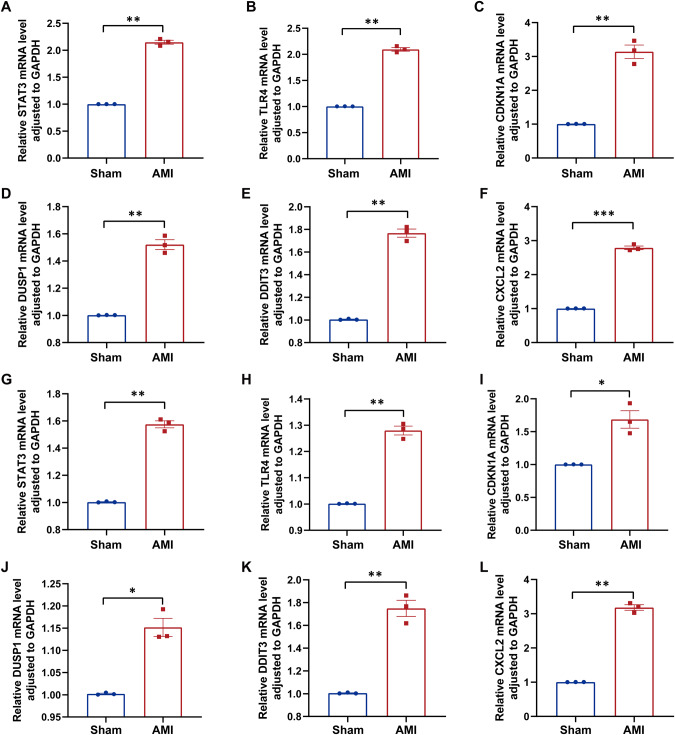
Genes expression in AMI and sham mouse cardiac tissues and blood evaluated by RT-qPCR at 24 h post-MI. mRNA levels of (**A**) STAT3, (**B**) TLR4, (**C**) CDKN1A, (**D**) DUSP1, (**E**) DDIT3, (**F**) CXCL2 in cardiac tissues. mRNA levels of (**G**) STAT3, (**H**) TLR4, (**I**) CDKN1A, (**J**) DUSP1, (**K**) DDIT3, (**L**) CXCL2 in blood. Significant differences between groups were determined using Student’s *t* test, data shown as mean ± SD, (*p < 0.05, **p < 0.01, ***p < 0.001, ****p < 0.0001).

**Fig. 8:**
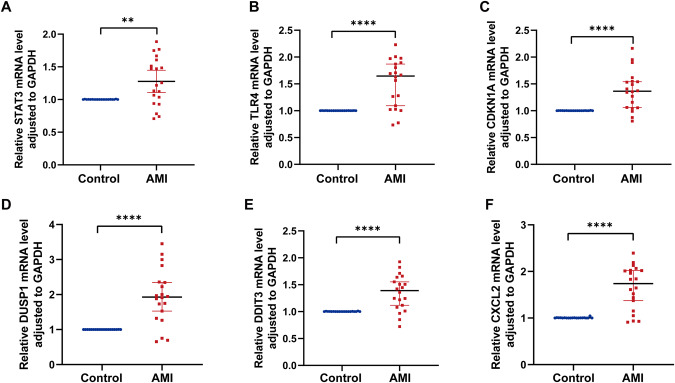
Genes expression in AMI patients and controls blood evaluated by RT-qPCR within 24 h post-MI. **A** STAT3, (**B**) TLR4, (**C**) CDKN1A, (**D**) DUSP1, (**E**) DDIT3, (**F**) CXCL2. Significant differences between groups were determined using Mann–Whitney test, data shown as median±95%CI, (*p < 0.05, **p < 0.01, ***p < 0.001, ****p < 0.0001).

In order to validate the expression of 6 feature genes in animals and patients. The AMI mouse model has been constructed, and a total of 20 patients with AMI and 20 healthy controls were enrolled in this study. In the AMI mouse heart tissue and peripheral blood, the RT-qPCR results have shown that the 6 feature genes (CDKN1A, DDIT3, STAT3, TLR4, CXCL2, DUSP1) were highly expressed at 24 h after AMI (Fig. 7). Similarly, the 6 feature genes were also significantly upregulated in the blood of most AMI patients when compared to healthy controls (Fig. 8). The baseline characteristics of AMI patients and healthy controls have been shown in Supplementary Table 2.

## Discussion

AMI is a leading cause of mortality among individuals with cardiovascular disease worldwide. Although thrombolytic and interventional treatments have improved prognosis, mortality rates remain high. Cardiac troponin I (cTnI) and cTnT are recommended biomarkers for AMI diagnosis, but their levels can be elevated in other myocardial injuries, limiting their specificity [31]. This complicates diagnosis and delays timely treatment, resulting in irreversible myocardial injury and poor prognosis. Therefore, identifying effective diagnostic biomarkers for precise prediction of AMI is crucial for improving therapy efficiency and patient outcomes.

Previous studies screening for diagnostic markers of AMI have mostly focused on ferroptosis- and immune-related genes [17, 32]. However, the pathogenesis of AMI is a particularly complicated process involving multiple biological pathways. A recent study has shown that cuproptosis is associated with the development and prognosis of AMI, suggesting a potential target for AMI treatment [19]. Intracellular copper accumulation is a significant cause of cuproptosis, which often occurs in cells with high metabolic activities, like mitochondria [33]. Therefore, we hypothesize that the cuproptosis involved in the development of AMI may be dependent on mitochondrial dysfunction [34]. Moreover, investigations have demonstrated that copper accumulation in the mitochondria is also connected to ferroptosis [20, 35]. This indicates a potentially complex relationship between ferroptosis and cuproptosis. There are currently no studies examining the link between cuproptosis and ferroptosis in AMI. We used bioinformatics tools to investigate the role of CFRGs in AMI as well as their interaction with immune infiltration, attempting to identify more precise AMI diagnostic markers.

In this study, we systematically screened 19 differential CFRGs in the peripheral blood of 74 healthy controls and 83 AMI patients. Functional enrichment analysis revealed that the differential CFRGs were enriched in the regulation of reactive oxygen species metabolic processes, inflammatory response-related pathways, and lipid and atherosclerosis, implying that differential CFRGs might participate in the initiation and progression of AMI through these biological processes. Most studies have demonstrated that inflammatory responses play an important role in the initiation and subsequent repair of myocardial infarction [36, 37]. This is in line with the results of our study. Then, we constructed the PPI network and screened 10 hub CFRGs (JUN, STAT3, MAPK14, TLR4, CDKN1A, DUSP1, DDIT3, ATM, CXCL2, SOCS1), all of which are potential candidate biomarkers closely related to AMI.

Immune cells are critical for maintaining cardiac homeostasis and repairing post-ischemic injury [38, 39]. Innate immune cells, such as macrophages and neutrophils, have been recruited into the heart after myocardial infarction, facilitating the clearance of necrotic cardiomyocytes and participating in the inflammatory response [40, 41]. Adaptive immune cells also participate in the immune response following AMI, but the underlying processes are still poorly understood. An observational study [42] indicated that after early coronary reperfusion, the number of CD8 cells in the blood was drastically reduced in STEMI cases, probably due to cell recruitment into the heart ischemic tissue. Our analysis shows that macrophages, neutrophils, and Tfh cells are highly expressed in the MI group, while CD8 + T cells, NK cells, Th1 cells, and TIL cells are lower. It was consistent with the previous studies. CCR has been demonstrated to modulate leukocyte infiltration and migration to inflammatory sites [43]. The CCL2-CCR2 axis is one of the classical CCR signaling pathways, which has contributed to regulating monocyte recruitment in the infarcted myocardium [44]. Several studies have shown that higher levels of CCL2 increase the risk of coronary artery disease and myocardial infarction [45]. In our study, we also demonstrated that the immune function of CCR was elevated in the AMI group. In the follow-up analysis, we identified the 6 hub CFRGs (STAT3, TLR4, CDKN1A, DUSP1, DDIT3, and CXCL2) most associated with immune infiltration as diagnostic feature genes through the Spearman correlation test. But the expression levels of all 6 feature genes were strongly positively linked with the CCR. This finding suggests that the 6 feature genes participate in the immunological and inflammatory responses of AMI, probably through CCR signaling pathways.

Then, we constructed a nomogram for predicting AMI using the 6 feature genes listed above, and the ROC curve showed good diagnostic efficacy with AUC areas of 0.805 and 0.94 for the training and validation datasets, respectively. This suggests that the 6-gene signature may have a good predictive value for AMI. These results were consistent with previous studies and validation using AMI mouse models and peripheral blood samples from AMI patients. During AMI, the upregulation of DDIT3, also known as CHOP, a pro-apoptotic transcriptional factor and a feature of endoplasmic reticulum (ER) stress, leads to increased apoptosis of myocardial cells [46, 47]. CHOP has been shown to have a close relationship with STAT3 in AMI. Li Y et al. [48] revealed that hypoxia-induced mitogenic factor (HIMF) can activate the CHOP-STAT3 signaling pathway, inhibiting M2 macrophage polarization and adversely regulating myocardial repair. However, another study suggested that upregulated P-STAT3 promotes anti-inflammatory M2 macrophage polarization, protecting against AMI [49]. CXCL2, a chemokine that recruits neutrophils, is elevated after acute myocardial infarction (AMI) and contributes to inflammation-mediated myocardial injury [50–52]. DUSP1, also known as MKP, is a MAPK phosphatase that dephosphorylates MAPKs. DUSP1-mediated dephosphorylation of JNK has been shown to have an antiapoptotic effect in myocardial ischemia-reperfusion (I/R) injury and has been identified as an immune-related diagnostic biomarker for AMI [32, 53, 54]. CDKN1A, also called p21, inhibits cardiomyocyte proliferation through acetylation modification in MI and has been proposed as a potential therapeutic target for MI [55, 56]. TLR4, a key pattern recognition receptor, is expressed on macrophages and cardiomyocytes and its elevated expression in MI exacerbates ischemic injury to cardiomyocytes [57], participating in multiple inflammatory signaling pathways [58–61]. These findings suggest that these 6 hub CFRGs may serve as diagnostic and therapeutic targets for AMI.

Finally, we predicted the miRNAs and candidate drugs that regulate the 6 feature genes. MiR-648 is predicted to regulate 3 feature genes (DDIT3, CXCL2, and STAT3) and has been linked to inhibiting tumor development and overcoming chemotherapy resistance [62]. But there are no studies about miR-648 in cardiac disease. MiR-4798-3p was indicated to modulate TLR4 and STAT3, and it was closely linked with the prevalence of atrial fibrillation in males and also could regulate genes involved in the pathogenesis of AF [63]. MiR-483-5p was found to be elevated in people with AF, which suggests that it could be used to predict AF [64], and it can exert neuroprotective effects after cardiopulmonary resuscitation by blocking ROS production and reducing MDA activity to attenuate oxidative stress injury [65]. Furthermore, studies have shown that miR-483-5p is associated with coronary plaque rupture and the formation of local thrombi, and its expression is significantly increased in both autoimmune myocarditis and acute myocardial infarction (AMI) [66, 67]. Moreover, it can serve as an independent predictor of AMI, but its specific mechanism of action in AMI requires further investigation.

In the current study, curcumin was shown to affect all 6 feature genes, and it has been discovered to have multiple effects, such as anti-inflammatory, anti-fibrosis, and so on. Liu Y et al [68], found that curcumin reduces cardiomyocyte injury generated by LPS by inhibiting the inflammatory response. Mahmoudi A et al [69], demonstrated that curcumin affects the process of liver fibrosis by impacting genes involved in extracellular matrix communication and oxidative stress. Although the function of the above-mentioned medicines and miRNAs in AMI is unclear, the particular pathways need further investigation. It also provides inspiration for the research and development of diagnosis and treatment related to AMI.

The pathophysiology of myocardial infarction is multifaceted, involving numerous biological processes. We posit that the crosstalk between ferroptosis, cuproptosis, and these biomarkers may be implicated in the pathogenesis of myocardial infarction. However, the precise regulatory mechanisms underlying this interplay require further investigation in future studies. While our discoveries are notable, this study is not without limitations, such as the limited number of cases and abbreviated follow-up period. As such, larger sample sizes and prolonged follow-up durations are imperative to better comprehend the prognostic relevance of these biomarkers for myocardial infarction. These limitations should be acknowledged in future investigations to yield fresh perspectives into the diagnosis and management of myocardial infarction.

## Conclusion

In this study, we identified a correlation between 175 ferroptosis-related genes and cuproptosis, with 19 genes showing differential expression in the AMI group compared to the controls, suggesting an interplay between cuproptosis and ferroptosis pathways in the development of myocardial infarction. In addition, using bioinformatics analysis, we screened 6 immune-related CFRGs as potential biomarkers for early AMI diagnosis. The ROC curve demonstrated their significant predictive value. Furthermore, the validation in both an AMI mouse model and peripheral blood samples from AMI patients confirmed the 6 feature genes, supporting their potential as biomarkers for AMI diagnosis.

## Supplementary information

supplementary material

supplementary table3

supplementary table4

## Data Availability

The datasets used in this research are available via public databases. The detailed introduction can be found in the paper/Supplementary Material.
