# Supplementary material for: Potential diagnostic biomarkers: 6 cuproptosis- and ferroptosis-related genes linking immune infiltration in acute myocardial infarction
Source: Genes Immun. 2023 Jul 8;24(4):159–70. doi: 10.1038/s41435-023-00209-8 (PMC10435388; doi:10.1038/s41435-023-00209-8)
Supplement: Supplementary file 1 — supplementary material [file 41435_2023_209_MOESM1_ESM.docx]

Supplementary Material

**Supplementary Table 1.** Primer sequences of 6 feature genes and 1 internal reference gene

| Primer(Homo sapiens) | Sequence（5’🡪3’） |
| --- | --- |
| GAPDH | F：AATGGGCAGCCGTTAGGAAA  R：GCCCAATACGACCAAATCAGAG |
| STAT3 | F：AGCAGTTTCTTCAGAGCAGGT  R：AGGCGTGATTCTTCCCACAG |
| TLR4 | F：GTCTGGCACCGTTTTAGT  R：TCCTGATTCTGCTCCTTCG |
| CDKN1A | F：TGCCGAAGTCAGTTCCTTGT  R：GTTCTGACATGGCGCCTCC |
| DDIT3 | F：CTGACCAGGGAAGTAGAGG  R：TGCGTATGTGGGATTGAG |
| CXCL2 | F：CAAACCGAAGTCATAGCC  R：GAACAGCCACCAATAAGC |
| DUSP1 | F: GGCCATTGACTTCATAGACTCCA  R: AACTCAAAGGCCTCGTCCAG |

| Primer(Mus musculus) | Sequence（5’🡪3’） |
| --- | --- |
| GAPDH | F：ACCCTTAAGAGGGATGCTGC  R：CCCAATACGGCCAAATCCGT |
| STAT3 | F：GACATTCCCAAGGAGGAGGC  R：TACGGGGCAGCACTACCT |
| TLR4 | F：TCCCTGCATAGAGGTAGTTCC  R：TCAAGGGGTTGAAGCTCAGA |
| CDKN1A | F：GCAGAATAAAAGGTGCCACAGG  R：GACAACGGCACACTTTGCTC |
| DDIT3 | F：TTGAAGATGAGCGGGTGGCAG  R：CACGTGGACCAGGTTCTCTCTC |
| CXCL2 | F：CCAAAAGATACTGAACAAAGGCA  R：CGAGGCACATCAGGTACGA |
| DUSP1 | F: CATCCCTGTGGAGGACAACC  R: CAGCATCCTTGATGGAGTCTATG |

**Supplementary Table 2**. Baseline clinical characteristics of the healthy controls and AMI patients

|  | Control | AMI | *P-*Value |
| --- | --- | --- | --- |
| Male, % | 12(60%) | 12(60%) | 1 |
| Age, years | 61.30±11.96 | 61.75±12.55 | 0.908 |
| BMI, kg/m^2^ | 24.89±1.94 | 24.61±2.89 | 0.721 |
| Smoking, % | 7(35%) | 8(40%) | 0.744 |
| Hypertension, % | 11(55%) | 13(65%) | 0.519 |
| Diabetes, % | 5(25%) | 9(45%) | 0.185 |
| Hyperlipidemia, % | 6(30%) | 8(40%) | 0.507 |
| LVEF, % | 57.25±2.75 | 48.45±9.19 | <0.001^***^ |
| Vessel disease |  |  |  |
| One, % |  | 3(15%) |  |
| Two, % |  | 7(35%) |  |
| Three, % |  | 10(50%) |  |
| Culprit vessel |  |  |  |
| LM, % |  | 3(15%) |  |
| LAD, % |  | 9(45%) |  |
| LCX, % |  | 3(15%) |  |
| RCA, % |  | 5(25%) |  |

Data are presented as number (%), mean ± SD. BMI, body mass index; LVEF, left ventricular ejection fraction; LM: left main coronary artery; LAD: left anterior descending artery; LCX: left circumflex artery; RCA: right coronary artery. ***, *P*< 0.001.


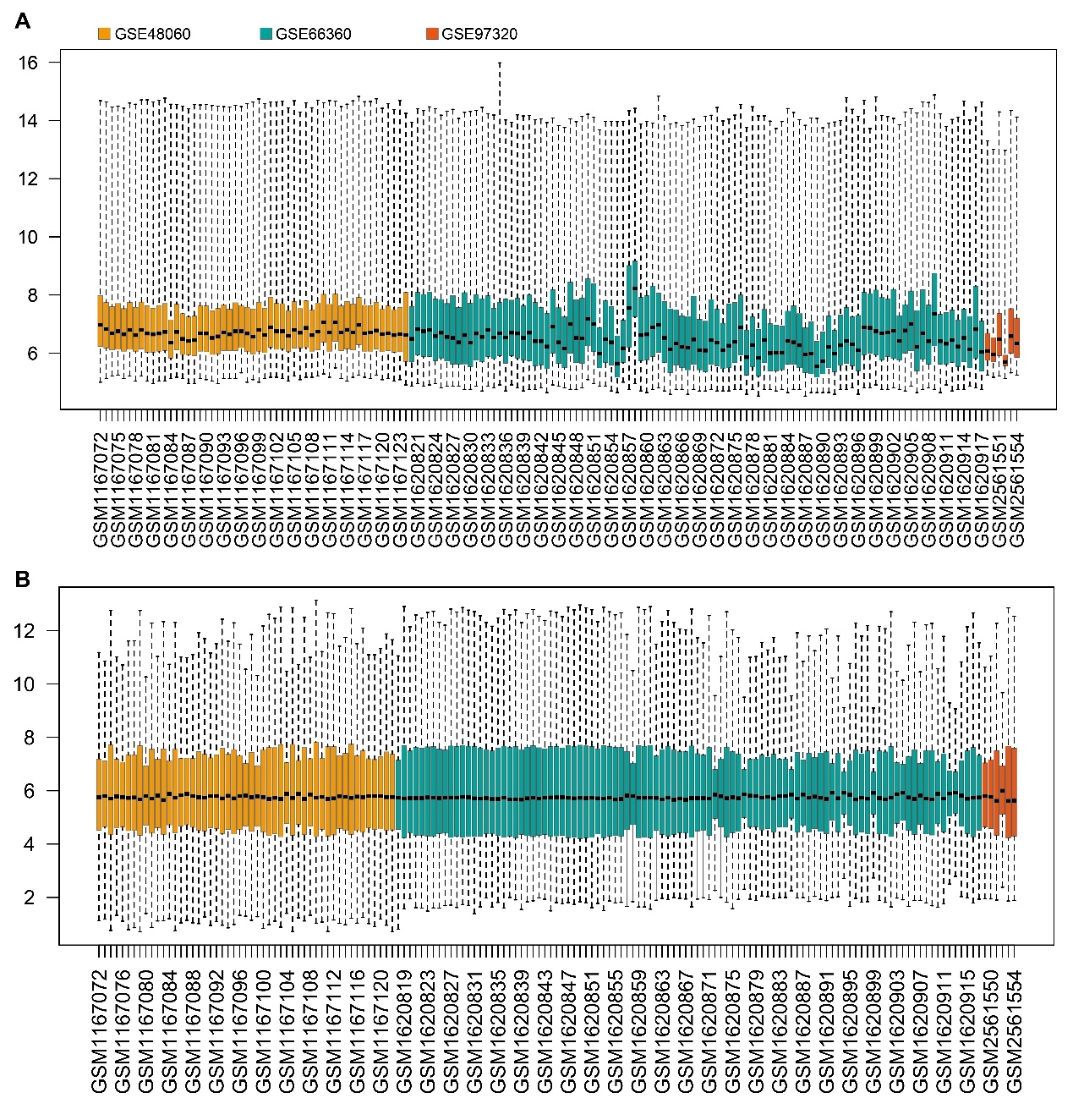


**Supplementary Figure 1. (A, B)** pre- and post-normalization of three expression datasets.


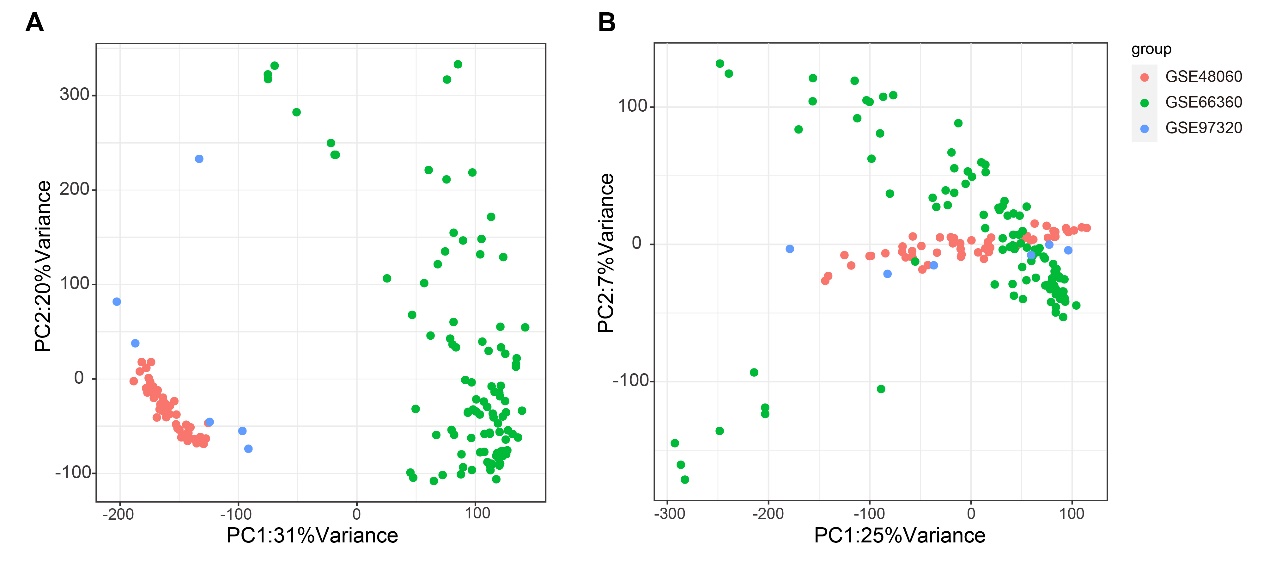


**Supplementary Figure 2. (A, B)** PCA diagrams of the chosen GEO datasets pre- and post- batch effect removal.
